# Ribosomal DNA internal transcribed spacer 2 sequence analysis and phylogenetic comparison of seven cockroach species in northwestern Iran

**DOI:** 10.1186/s13104-019-4089-3

**Published:** 2019-01-23

**Authors:** Mostafa Farmani, Hamidreza Basseri, Behzad Norouzi, Saber Gholizadeh

**Affiliations:** 1 Cellular and Molecular Research Center, Urmia University of Medical Sciences, Urmia, Iran; 2 Medical Entomology Department, School of Public Health, Urmia University of Medical Sciences, Urmia, Iran; 3 Medical Entomology Department, School of Public Health, Tehran University of Medical Sciences, Tehran, Iran; 4 Research Center of Health and Environment, Guilan University of Medical Sciences, Rasht, Iran

**Keywords:** rDNA-ITS2, Phylogeny, Blattidae, Ectobiidae, Corydiidae

## Abstract

**Objectives:**

The current study was conducted to identify cockroach species (Blattodea) of northwestern Iran in public places using morphological characteristics and ribosomal DNA internal transcribed spacer 2 (rDNA-ITS2). Sequences were analyzed with Basic Local Alignment Search Tool (BLAST) searches, Neighbor-Joining methods based on and Tamura-Nei phylogenetic analyses. In addition, eight cockroach rDNA-ITS2 sequences from China, India, Iran and the United States obtained from GenBank were compared to those obtained in this study.

**Results:**

Specimens collected in Iran were identified as *Periplaneta americana* (L.), *Shelfordella lateralis* (Walker), *Blatta orientalis* (L.) (Blattodea: Blattidae), *Blattella germanica* (L.), *Supella longipalpa* (F.) (Blattodea: Ectobiidae), *Polyphaga aegyptiaca* (L.), and *Polyphaga saussurei* (Dohrn) (Blattodea: Corydiidae). rDNA-ITS2 nucleotide sequence analysis showed 100% similarity between *P. aegyptiaca* and *P. saussurei* species collected from Iran despite morphological differences. However, ITS2 sequence of *P. americana* submitted from China showed 30.49–31.71% difference to *P. americana* sequences from Iran and the United States. The results highlight the importance of morphological identification of cockroach species before conducting molecular techniques.

## Introduction

Cockroach is one of the most important urban pests in the world. They are mostly known for their role in allergies and can transmit some diseases to human [1–4].

Mirzayans reported 24 species of cockroaches in Blattidae, Ectobiidae, and Corydiidae families in Iran [5]. More recent surveys by Hanafi-Bojd, Sadaghiyani and Hashemi-Aghdam, Oshaghi reported 3 families, 14 genera, and 26 species in Iran [6, 7]. Two more species are *Parcoblatta* sp. [8], and *Polyphaga* sp. [7].

Mitochondrial and nuclear molecular markers are used for the precise identification of cockroach species and their phylogenetic relationship [9]. rDNA-ITS2 might be appropriate for mushrooms and Diptera, but it does not mean that it is appropriate for (some) cockroaches [10–12]. Ribosomal DNA has not been used frequently for identification of cockroach species [13]. ITS2 length varies among different cockroach species [13], and ITS2 sequences are often more polymorphic between species than within species [14]; therefore, it could be useful for molecular identification of sibling species [15].

A better understanding of the cockroach ecology and taxonomy is essential for the successful pest control program. Thus, a routine survey of the cockroach population in northwestern Iran will greatly contribute to the success of the control program. This study was designed to identify morphologically and molecularly cockroach species in northwestern Iran and to determine the phylogenetic relationships (using rDNA-ITS2 sequences) among these species. New set of primers were designed as universal primers to amplification of rDNA-ITS2 fragment in cockroach species.

## Main text

### Methods

#### Cockroach collection and morphological identification

The majority of cockroaches used in this study were collected manually by searching their shelters using flashlight at night from 30 locations in Urmia, Iran (37°33′19″N 45°04′21″E) in 2013–2015. Plastic bottle traps and sticky cards (n = 10 in each location) baited with sugar, biscuits and dried breads were used to collect cockroaches in houses, hospitals, dormitories and landfill. Trappings were conducted from 6:00 p.m. to 6:00 a.m. in 2015. Cockroaches were captured from the plastic bottle traps and transferred to containers individually. The inner surface of the plastic bottle traps was coated with butter to prevent escape.

Adult cockroaches were killed at − 20 °C and identified using morphological keys [5, 6]. Voucher specimens were deposited in the Entomology Laboratory at the School of Public Health (SPH), Urmia Medical Sciences University (UMSU), Urmia, Iran.

#### DNA extraction

Genomic DNA was extracted from the thorax of individual cockroach species, stored in 70% ethanol, using YTA Genomic DNA Extraction Mini Kit (Yekta Tajhiz Azma, Tehran, Iran). Based on manufacturer’s instruction, a 25-mg tissue sample was removed from the thorax of each cockroach with a surgical blade and homogenized in 200 µL TG1 buffer by grinding with a micropestle containing liquid nitrogen. After adding 20 µL proteinase K, the mixture was incubated at 60 °C for 1–2 h. 200 μL of TG2 buffer was added and re-incubated for 10 min at 70 °C, after which 200 μL cold ethanol was added. The mixture was transferred to TG Mini Column and centrifuged for 1 min at 8000 rpm. The flow-through was discarded and TG Mini Column was transferred to a new Collection Tube. DNA was washed with 500 μL of W1 and 750 μL of wash buffers by centrifuging for 1 min at 14,000 rpm. Total DNA was eluted to the elution tube by adding 100 μL elution buffer or ddH_2_O (pH 7.5–9.0) and stored at 4 °C or − 20 °C until use [16].

#### Primer designing and PCR amplification

Cockroach-specific primers (5.8S TGGGTCGATGAAGAACGC and 28S ATTCAGCGGGTAGTCTCG) were designed based on cockroach rDNA sequences available in the GenBank (GenBank ID: AF005243, KF899831, and EU306665) using the softwares Gene Runner (Hastings Software Inc. 1994) and Standard Nucleotide BLAST [17].

PCR reactions of ITS2 fragment were performed in a total volume of 25 µL master mix. Each reaction contained 2 µL genomic DNA, 12.5 μL PCR Master Mix (Yekta Tajhiz Azma, Tehran, Iran), 0.2 μL Taq polymerase, 1 μL each primer (forward and reverse), and 8.3 μL ddH_2_O. The PCR amplification profile was set as follows: initial template denaturation at 95 °C for 5 min, followed by 30 cycles of denaturation at 95 °C for 1 min, annealing at 54 °C for 1 min, and extension at 72 °C for 1 min, with a final 10-min elongation step at 72 °C. The PCR products (5 μL) were run on 1.5% agarose gel stained with safe stain (Yekta Tajhiz Azma Co. Cat No. YT0001, Tehran, Iran), and bands were visualized by UV trans-illumination (Syngene GBOX/EF, Cambridge, England). A total of 30 specimens were subjected to sequencing with an ABI-377 automatic sequencer (SeqGen, Torrance, Canada), using the same amplification primer.

Eight rDNA-ITS2 sequences of *Blatta orientalis* L. (GenBank IDs: EU306665, KF899833), *Periplaneta americana* (L.) (KF899831, AF321248, and AJ577262) and *Blattella germanica* L. (KF899832, AF005243, and AF321244) from China [18], India [19], central Iran [20] and the United States [13], belong to seven species collected in the current study, were also obtained from GenBank. These sequences and 30 sequences of the seven Iranian cockroach species were aligned, and a *Mantis religiosa* L. (Mantodea: Mantidae) ITS2 sequence (GenBank ID: AY859585) [21] was used as out-group. The sequences obtained from GenBank were compared to the sequences obtained in this study.

#### Sequence analysis

The acquired ITS2 sequences were annotated according to the previously submitted sequences using the ITS2 annotation tool, version 3.0.13 [22]. For rDNA-ITS2 sequence alignment and phylogenetic analyses two online software programs, BLAST [23], Clustal Omega [24], and an offline software, Molecular Evolutionary Genetics Analysis 7 (MEGA7) [25], were utilized.

The phylogenetic tree was constructed using both maximum likelihood and neighbor-joining methods based on Tamura-Nei model [26, 27]. The percentages of replicating trees in which the associated taxa clustered together in the bootstrap test (1000 replicates) are indicated for each branches [28].

### Results

Three-hundred-twenty-one cockroaches representing 6 genera and 7 species were collected in the current study (Table 1). *Blattella germanica* was the most frequently collected species, whereas *Polyphaga aegyptiaca* L. and *Polyphaga saussurei* Dohrn were the least frequently collected.

**Table 1:** Species and frequency of cockroaches collected from Urmia, West Azerbaijan Province, Iran

Family	Species	Number collected (% total collected)
Blattidae	*Blatta orientalis* L.	23 (7.2)
	*Shelfordella lateralis* Walker	62 (19.3)
	*Periplaneta americana* L.	57 (17.8)
Ectobiidae	*Supella longipalpa* F.	19 (5.9)
	*Blattela germanica* L.	146 (45.5)
Corydiidae	*Polyphaga aegyptiaca* L.	7 (2.2)
	*Polyphaga sausserei* Dohrn	7 (2.2)

The amplified fragment size using designed primers was 340 bp in *Supella longipalpa* (F.), 385 bp in *P. aegyptiaca* and *P. saussurei,* 412 bp in *P. americana* and *S. lateralis*, 418 bp in *B. orientalis*, and 592 bp in *B. germanica*. Results from ITS2 annotation tool showed different ITS2 sizes, varying from 179 bp in *S. longipalpa* to 431 bp in *B. germanica*. Also, the 128-bp upstream and 33-bp downstream of ITS2 sequences were 5.8 s and 28 s, respectively. These sequences were submitted to GenBank under the accession numbers KY817789 to KY817818, representing the first records for some species in Iran and the world.

The numbers of cockroach rDNA-ITS2 sequences deposited in GenBank were low, which limited our ability to conducted more BLAST analyses and comparisons to species collected in Iran. Sequence similarity within species of *B. germanica*, *S. lateralis*, *B. orientalis*, *S. longipalpa*, *P. aegyptiaca*, and *P. saussurei*, collected from Urmia, was 99.55–100%, whereas the identity within *P. americana* sequences was 97.61–100%. BLAST comparison of *B. germanica* sequences from Iran and the United States showed 99.53–100% similarity. The sequence of *B. orientalis* from India was only 45.28% similar to those isolated from *B. orientalis* collected in this study, but showed more similarity (98.36–98.83%) to those of *B. germanica* from GenBank (KF899832, AF005243, and AF321244). The similarity between sequences of *P. americana* from Iran (KF899831) and the United States (AF321248) was 97.21–100%, but the similarities of the Iranian and American sequences to the Chinese sequence (AJ577262) were 68.70% and 68.29–69.51%, respectively. Moreover, the sequence sizes of rDNA-ITS2 region in *P. americana* (from Iran, the United States, and China) and *P. saussurei* (from Iran) were the same as *S. lateralis* (from Iran) and *P. aegyptiaca* (from Iran), i.e. 251 bp and 224 bp, respectively. Sequence similarity analysis showed 88.80% identity between *P. americana* and *S. lateralis*, whereas it was 99.55% in *P. saussurei* and *P. aegyptiaca*.

**Fig. 1:**
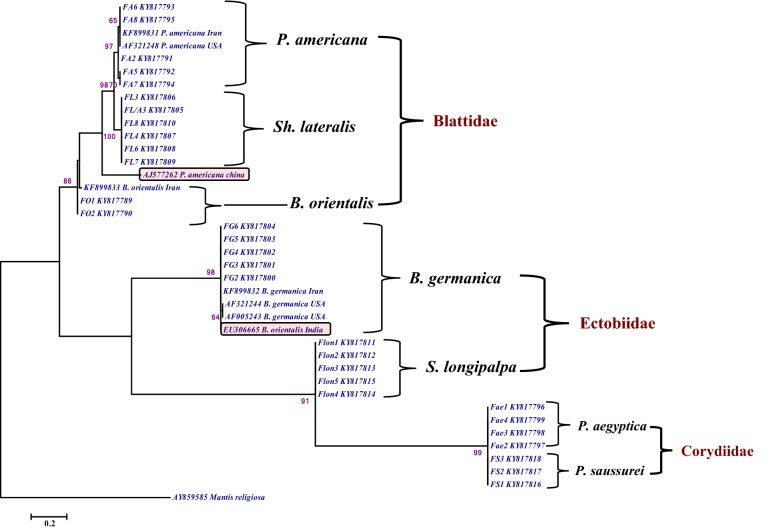
Molecular phylogenetic analysis of 30 Iranian cockroach rDNA-ITS2 sequences based on Maximum Likelihood method. The bootstrap consensus tree inferred from 1000 replicates is taken to represent the evolutionary history of the taxa analyzed. Bootstrap values < 50% were deleted. All positions containing gaps and missing data were eliminated from the dataset complete deletion option. *Mantis religiosa* was used as out-group

Phylogenetic analysis revealed that the seven Iranian cockroach species were clustered into two main branches (Fig. 1). *Periplaneta americana*, *B. orientalis* and *S. lateralis*, members of Blattidae, were clustered in the first branch. However, *B. germanica* and *S. longipalpa* were placed in two separate plural. *Polyphaga aegyptiaca* and *P. saussurei* were clustered in the same clade and branch (Fig. 1). Interestingly, *B. orientalis* from India (EU306665) was placed in the *B. germanica* cluster, whereas *P. americana* from China (AJ577262) was clustered in a separate clade near Blattidae.

### Discussion

The cockroach fauna in Urmia is unknown, it is not included in Iranian checklist of cockroaches [5, 7].

Several sequences of species obtained in this study were not completely similar to those of the same species obtained from GenBank. The sequence of *B. orientalis* from India (EU306665) was similar to that of *B. germanica* obtained in this study. While, these two species belongs to two different cockroach families with high distinct in morphology, rDNA-ITS2 size and sequence. Phylogenetic analysis also revealed that sequence of *P. americana* from China (AJ577262) was clustered in a separate clade from *P. americana* from Iran and the United States. It seems those are morpho-taxonomic mistakes in cockroach species identification. These mistakes suggested it is good advice to ensure cockroach species identification before any molecular analysis and depositing the sequences to the GenBank.

This study showed that rDNA-ITS2 sequence analysis of *P. aegyptiaca* and *P. saussurei* is 100% similar in size (224 bp) and sequence. A high degree of rDNA-ITS2 sequence and size similarity, despite morphological differences in body shape and color, also had been reported between *Anopheles hyrcanus* (Pallas) and *Anopheles pseudopictus* (Grassi) (Diptera: Culicidae) (Ponçon et al. 2008). Such similarity suggested that *P. aegyptiaca* and *P. saussurei* may be the same species although they are considered separate species based on morphological characteristics. However, only ITS2 marker is not sufficient for these conclusion and need to study with other molecular markers.

In the phylogenetic tree, three Iranian cockroach families were classified into four clades. It was notable that *B. germanica* and *S. longipalpa* belongs to Ectobiidae clustered in two different clades. Recent phylogenetic analyses of cockroaches showed that Ectobiidae are paraphyletic [29–31].

### Conclusions

We would like to emphasize the importance of considering both morphological and molecular data in identifying field collected specimens of cockroaches before designing, implementing and evaluating control programs. Incorrect identification of insect species may sometimes lead to the application of incorrect vector control strategies [32] and can have significant consequence to the efficiency of vector and urban pest management programs.

## Limitations

The major concerns within this study is the use of only one genetic marker (ITS2) instead of multiple markers such as cytochrome oxidase I (COI) and cytochrome oxidase II (COII) (because of founding limitations for MSc student project). The current study were conducted in Urmia district among seven cockroach species, however, the efficiency of new primers need to evaluate on other cockroach species in large areas.

## Abbreviations

rDNA-ITS2: ribosomal DNA internal transcribed spacer 2
BLAST: Basic Local Alignment Search Tool
MEGA7: Molecular Evolutionary Genetics Analysis version 6.0
EMBL: European Molecular Biology Laboratory
DDBJ: DNA Data Bank of Japan
COI: cytochrome oxidase I
COII: cytochrome oxidase II
